# Standardizing Cranioplasty Outcomes Following Stroke or Traumatic Brain Injury: Protocol for the Development of a Core Outcome Set

**DOI:** 10.2196/37442

**Published:** 2023-04-17

**Authors:** Harry Mee, Ana M Castaño-Leon, Ivan Timofeev, Amos Adeleye, Indira Devi Bhagavatula, Niklas Marklund, Susanne Muehlschlegel, Katie Bond, Clare Clement, Kirsty Grieve, Nicola Owen, Gemma Whiting, Carole Turner, Andres Mariano Rubiano Escobar, Dhaval Shukla, Maria Paul, Judith Allanson, Valerie Pomeroy, Edoardo Viaroli, Elizabeth Warburton, Adam Wells, Gregory Hawryluk, Adel Helmy, Fahim Anwar, Stephen Honeybul, Peter Hutchinson, Angelos Kolias

**Affiliations:** 1 Division of Rehabilitation Medicine and Neurosurgery Department of Clinical Neurosciences Cambridge University Hospital NHS Foundation Trust Cambridge United Kingdom; 2 Division of Rehabilitation Medicine Department of Clinical Neurosciences Cambridge University Hospital NHS Foundation Trust Cambridge United Kingdom; 3 NIHR Global Health Research Group on NeuroTrauma University of Cambridge Cambridge United Kingdom; 4 Department of Neurosurgery Instituto de Investigación Sanitaria Hospital 12 de Octubre Hospital Universitario 12 de Octubre Madrid Spain; 5 Division of Neurosurgery Department of Clinical Neurosciences Cambridge University Hospital NHS Foundation Trust Cambridge United Kingdom; 6 University of Ibadan Ibadan Nigeria; 7 Department of Neurosurgery National Institute of Mental Health and Neurosciences Bangalore India; 8 Department of Clinical Sciences Lund Neurosurgery Lund University, and Skane University Hospital Lund Sweden; 9 University of Massachusetts Medical School Massachusetts, MA United States; 10 Bristol Trials Centre Bristol Medical School Bristol United Kingdom; 11 Department of Neurosciences and Neurosurgery Valle Salud IPS (Health Service Provider Institutions) Network Cali Colombia; 12 Adelaide University Adelaide Australia; 13 Division of Anaesthesia University of Cambridge Cambridge United Kingdom; 14 Neurorehabilitation Department University of East Anglia Norwich United Kingdom; 15 Division of Stroke Medicine Department of Clinical Neurosciences Cambridge University Hospital NHS Foundation Trust Cambridge United Kingdom; 16 Department of Neurosurgery at the University of Adelaide University of Adelaide Adelaide Australia; 17 Section of Neurosurgery University of Manitoba Manitoba, MB Canada; 18 Department of Neurosurgery Royal Perth Hospital Perth Australia

**Keywords:** cranioplasty, TBI, stroke, core outcome set, domains, COMET, health Interventions, recovery, traumatic brain injury, neurological, neurology, reporting, outcome

## Abstract

**Background:**

Core outcome sets (COSs) are important and necessary as they help standardize reporting in research studies. Cranioplasty following traumatic brain injury (TBI) or stroke is becoming increasingly common, leading to an ever-growing clinical and research interest, especially regarding the optimal material, cost-effectiveness, and timing of cranioplasty concerning neurological recovery and complications. Consequently, heterogeneous reporting of outcomes from such diverse studies has led to limited meta-analysis ability and an ongoing risk of outcome reporting bias. This study aims to define a standardized COS for reporting in all future TBI and stroke cranioplasty studies.

**Objective:**

This study has four aims: (1) undertake a systematic review to collate the most current outcome measures used within the cranioplasty literature; (2) undertake a qualitative study to understand better the views of clinicians, patients' relatives, and allied health professionals regarding clinical outcomes following cranioplasty; (3) undertake a Delphi survey as part of the process of gaining consensus for the COS; and (4) finalize consensus through a consensus meeting resulting in the COS.

**Methods:**

An international steering committee has been formed to guide the development of the COS. In addition, recommendations from other clinical initiatives such as COMET (Core Outcomes and Effectiveness Trials) and OMERACT (Outcome Measures in Rheumatology) have been adhered to. Phase 1 is data collection through a systematic review and qualitative study. Phase 2 is the COS development through a Delphi survey and consensus meetings with consensus definitions decided and agreed upon before the Delphi survey begins to avoid bias.

**Results:**

Phase 1 started at the end of 2019, following ethical approval in December 2019, and the project completion date is planned for the end of 2022 or beginning of 2023.

**Conclusions:**

This study should result in a consensus on a COS for cranioplasty, following TBI or stroke, to help standardize outcome reporting for future studies, which can be applied to future research and clinical services, help align future studies, build an increased understanding of cranioplasty and its impact on a patient’s function and recovery, and help standardize the evidence base.

**International Registered Report Identifier (IRRID):**

DERR1-10.2196/37442

## Introduction

### Background

Several randomized trials [1,2] have shown that decompressive craniectomy, which is a surgical procedure in which a large section of the skull is removed to accommodate brain swelling, can be helpful in the management of patients with substantial brain swelling and raised intracranial pressure after a traumatic brain injury (TBI) or stroke. Patients who survive usually have their skull reconstructed a few months later through an operation termed cranioplasty. Cranioplasty not only restores skull integrity, providing a degree of mechanical protection to the brain, but can also improve neurological function [3]. However, there are various interesting and unanswered clinical questions around cranioplasty, including complication rates, neurological recovery and outcomes, and the influence timing has on these [4], material choice, and overall cost-effectiveness. As a result, there are many studies published in the literature exploring these themes [3,5,6]. This has led to variability of the terminology used and heterogeneity of outcomes and outcome measures used. These aspects pose substantial barriers to establishing an evidence-based approach to clinical care and research for cranioplasty. Nevertheless, the development of common data elements (CDEs) and core outcome sets (COSs) is becoming increasingly common to overcome such obstacles.

The importance of high-quality, evidence-based clinical practice is known to all, as without reliable, cost-effective interventions and treatments, clinicians' decision-making can become compromised. A core outcome set (COS) is defined as “an agreed standardized set of outcomes that should be measured and reported, as a minimum, in all clinical studies and trials in specific areas of health or health care” [7]. They are a strategy to help as they result in predefined, consensus-derived outcomes that should be reported on in any given condition. This can improve the quality of evidence by allowing studies to be combined for systematic reviews, meta-analyses, and clinical guidelines [8]. The concept was first developed in the early 1990s with the OMERACT (Outcome Measures in Rheumatology) group, which started by creating a COS for rheumatoid arthritis trials and now has a widely used methodological framework on which to identify and validate such sets [9]. Over the past 10 years, further initiatives, including the COMET (Core Outcome Measures in Effectiveness Trials) and the International Consortium for Health Outcomes Measurement, have also produced frameworks as a base for the development of a COS in a specified disease or health condition.

**Figure 1:**
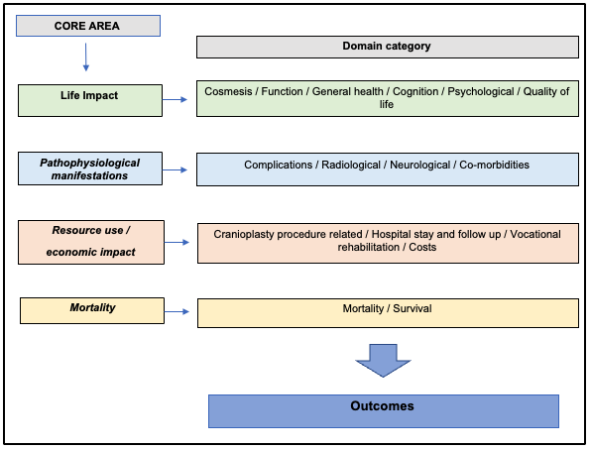
Cranioplasty outcome domains and associated core area categorization using the OMERACT (Outcome Measures in Rheumatology) 2.0 filter.

Conceptual models have been developed for studying health, diseases, and outcomes, but not all are appropriate for direct use in COS development [10]. The OMERACT initiative overcame this issue by broadening the scope of one of these frameworks, the International Classification of Functioning, Disability, and Health, by incorporating the Wilson and Cleary [11] model of health-related quality of life and thus developing what is now in its second iteration: the OMERACT Filter 2.0 [12], which is widely used as a framework primarily for COS development and has “4 core areas” broadly representing important categories of outcomes measurable in clinical trials (Figure 1). The core areas are “life impact,” “resource use and economic impact,” “pathophysiological manifestations,” and “death*.”* The core areas are defined, with domains of the specified health-related condition grouped within one of the core areas. For example, the life-impact core area can include domains from both the International Classification of Functioning, Disability, and Health and health-related quality-of-life frameworks. Resource use and economic impact should have domains describing the economic impact of the health condition for an individual and society and specific resource use. Finally, the pathological manifestation’s core area is to assess whether the effect of the intervention targets the pathophysiology of the health condition explicitly. It is recommended that at least one domain from each core area is included in the COS [12].

### Study Aims and Objectives

#### Aim

The aim is to develop a COS for cranioplasty research following TBI or stroke by defining a standardized and agreed set of outcomes that should be reported as a minimum [13] while adhering to the COS-STAD (Core Outcomes Set-Standards for Development) recommendations [14] for COS development (Textboxes 1 and 2).

**Cranioplasty core outcome set scope.**

**Health condition**

- Patients who have undergone a decompressive craniectomy secondary to a traumatic brain injury or stroke await a cranioplasty

**Population**

- Adult patients older than 16 years

**Health intervention**

- Cranioplasty

**Context**

- The core outcome set will be used in future cranioplasty clinical studies and clinical trials to inform clinical decision-making

**Study protocol definitions, adapted from the OMERACT (Outcome Measures in Rheumatology) filter 2.0.**

**Health**

- A state of complete physical, mental, and social well-being and not merely the absence of disease or infirmity.

**Health condition**

- A situation of impaired health.

**Health intervention**

- An activity performed by, for, with, or on behalf of a client(s) whose purpose is to improve individual or population health, alter or diagnose the course of a health condition, or improve functioning.

**Core area**

- An aspect of health or a health condition needs to be measured to assess a health intervention's effects appropriately.

**Domain**

- Component of core area: a concept to be measured, a further specification of an aspect of health, categorized within a core area.

**Outcome**

- Any identified result in a domain arising from exposure to a causal factor or a health intervention.

**Measurement instrument**

- A tool to measure the quality or quantity of a variable in a domain or a contextual factor. The tool can be a single question, a questionnaire, a score obtained through physical examination, a laboratory measurement, or a score obtained through observation of an image.

**Outcome measurement instrument**

- A measurement instrument was chosen to assess the outcome.

**Core domain set**

- For studies of health interventions, the minimum set of Domains and Subdomains is necessary to cover all Core Areas adequately, that is, sufficiently measure all relevant concepts of a specific health condition within a specified setting.

**Core outcome measurement set**

- The minimum set of outcome measurement instruments must be administered in each intervention study of a particular health condition within a specified setting to adequately cover a corresponding Core Domain Set.

**Scope**

- The set of factors describes the studies and circumstances to which the core outcome measurement set will apply.

**Contextual factor**

- A variable that is not an outcome of the study must be recognized (and measured) to understand the results.

#### Objectives

This study has 4 objectives:

- Undertake a systematic review to collate the most current outcome measures used within the cranioplasty literature
- Undertake a qualitative study to understand better the views of clinicians, patients’ relatives, and allied health professionals regarding clinical outcomes following cranioplasty
- Undertake a Delphi survey as part of the process of gaining consensus for the COS
- Finalize consensus through a consensus meeting resulting in the COS

#### Study Design

**Figure 2:**
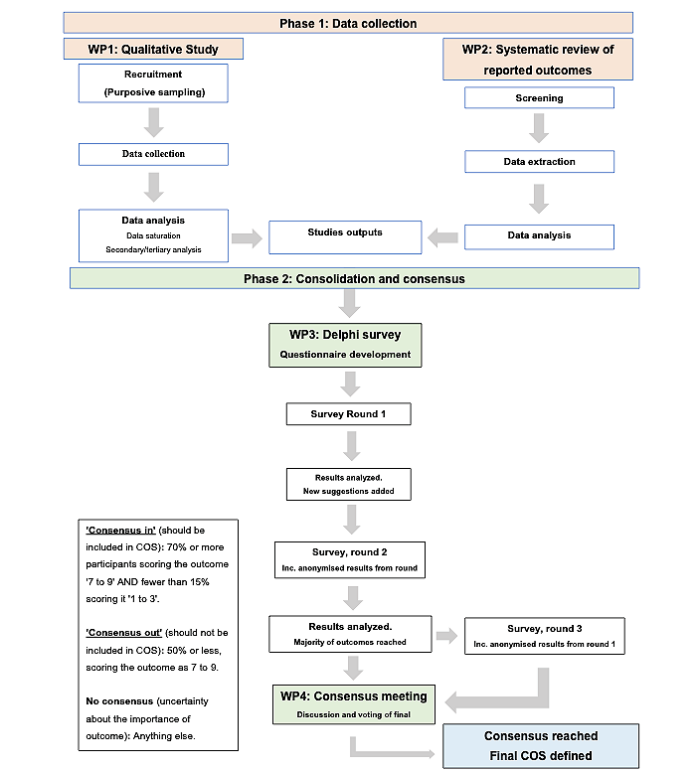
Study Flowchart. COS: Core Outcome Set.

This is a mixed methods study and will be divided into 2 main phases (Figure 2):

- Phase 1 (information gathering):
  WP1: systematic review
  WP2: a qualitative study producing a comprehensive list of outcome domains relating to the cranioplasty pathway

- Phase 2 (consolidation and consensus):
  WP3: Delphi study
  WP4: consensus meeting and finalization of COS

#### Stakeholder Involvement

Advocates from multiple stakeholder groups were a part of the COS development [13,15] by COS methodologists. Adhering to this, the COAST study has 4 stakeholder groups:

1. Patients and/or relatives: people who have had or are awaiting a cranioplasty or their relatives
2. Surgeons: experts who perform cranioplasty in their clinical practice
3. Physicians (nonsurgeons) and intensivists: physicians who care for and treat patients awaiting or having had a cranioplasty
4. Nurses, allied health professionals, and researchers: professionals who treat and help rehabilitate those patients who have had or are awaiting a cranioplasty or have a research interest

These 4 stakeholder groups represent the key personnel in the acute and long-term management and rehabilitation of patients who have undergone or will undergo a cranioplasty. The groups will provide a wealth of experience and views from all aspects of the patient’s care pathway.

#### COS Development Team

An international steering committee has been formed to oversee the development of the COS. The committee represents a broad range of disciplines and regions of the world, all with clinical interest in and involvement with cranioplasty from a research perspective. A project team oversees the COS development process’ day-to-day management, which includes 3 steering committee members and a coordinator. In addition, the project team will establish the methodology and address the key aspects of the project. Key definitions and concepts used to develop this COS have stemmed from the OMERACT initiative (Textbox 2).

## Methods

### Phase 1: Information Gathering

#### WP1: Systematic Review

Which outcome domains are used within the published literature for patients undergoing a cranioplasty following TBI or stroke and their recovery and rehabilitation?

#### Participants/population

Participants should be adult patients aged >16 years who have undergone a decompressive craniectomy.

#### Intervention

The intervention being studied will be cranioplasty.

#### Identification of Studies

A search of three databases (PubMed, Embase, and Web of Science) from 1990 to the present analysis will be undertaken to identify either prospective cohort studies with >10 cranioplasty patients or retrospective cohort studies with >20 cranioplasty patients, and two reviewers (AMCL and HM) will independently assess the abstracts of the studies resulting from the search.

#### Eligibility of Studies

Two reviewers (ACL and HM) will independently review the full texts of the screened abstracts, with any disagreements for inclusion being resolved through discussion. If required, a third reviewer (AK) will be consulted.

#### Data Extraction

Data will be extracted independently by two reviewers (AMCL and HM). The data will then be reviewed to ensure all outcomes have been identified and no unresolved issues arise. Any disagreements will be resolved through discussion with a third reviewer (AK) available for consultation if required.

#### Outcomes

The included studies’ outcomes will be categorized into domains under one of the four core areas of the OMERACT 2.0 filter framework [12]. Outcomes will be classified as primary when explicitly identified by the study authors, and all other outcomes will be classified as secondary. Each core area and subsequent domain evaluation of the number and frequency of the different outcomes will be analyzed.

#### WP2: Stakeholder Consultation—A Qualitative Study

To ensure a comprehensive list of outcomes, a qualitative study involving participants in the 4 stakeholder groups will be run, exploring cranioplasty experiences and services and discussing which outcomes are essential to the individual or group. These will then be combined with the outcomes from WP1 to develop the Delphi survey questionnaire.

The qualitative study will include both interviews and focus groups. Participants will be sampled purposively, and the sample size will be guided by data saturation, with 25 and 40 participants across the stakeholder groups likely to be required to reach saturation. A thematic analysis [16] will be used using deductive and inductive coding to analyze known topics of interest and generate new information as the study proceeds. Disconfirming evidence and outlying data will be searched to enhance the reliability and rigor of the analysis process and findings [17]. Data will be compared across and within groups. Qualitative data analysis software (ATLAS.ti) [18] will facilitate data management and analysis.

### Phase 2: Consolidation and Consensus

#### WP3: Delphi Survey

The survey will be online using the Delphi Manager software developed by COMET at the University of Liverpool. Participants will be asked to register and assign themselves to one of the four stakeholder groups. Registration will be implied consent.

#### Questionnaire Development

This will be drawn from outcomes collated in phase 1 (WP1 and 2) and reviewed and signed off by the steering committee before use.

#### Recruitment and Sample Size

This will involve centrally coordinating with phase 1 participants invited to participate in further recruitment by post, email, or in person. Participants will also be advised of the importance of completing the Delphi survey, and email reminders will be generated as required to help with compliance.

There are no specific guidelines for the optimal number of participants in a Delphi survey, with previous COS studies having reported 140-150 participants in total [19,20]. Therefore, a pragmatic approach will be taken with the number of participants recruited for each stakeholder group. No new recruitment will occur after the completion of round 1.

#### Rounds and Scoring

The survey is an iterative process, with a minimum of two rounds but occasionally requiring a third or fourth. All participants score all outcomes using a 9-point Likert scale [21] (Table 1). There will be an “unable to score” option, allowing participants to abstain from scoring a particular outcome if they feel they do not have the expertise to do so; this has been demonstrated to work well in previous COS development projects [22]. A comments box will accompany each outcome to allow feedback as required.

Between rounds, scores are analyzed, summarized, and fed back to all participants in a histogram displaying the distribution of the scores of each outcome, with their scores highlighted. In addition, if there are any descriptive comments for a particular outcome, these will be summarized and presented.

**Table 1:** Likert scoring scale.

Likert scale	Outcome
1-3	Limited importance
4-6	Important but not critical
7-9	Critical
Unable to score	N/A^a^

^a^N/A: not applicable.

All outcomes from round 1 will be carried forward to round 2, with any additional recommended outcomes also included in round 2. Participants who completed round 1 in the time allocated will be asked to participate in round 2. Each participant will be shown all the outcomes and scores, the number of respondents, the distribution of respondents' scores, and their scores from round 1. The participant will then be asked to rescore each outcome measure with the feedback from round 1 but categorized by stakeholder group. Brookes et al [21] have shown that this feedback method may improve overall agreement in scoring because it enables reflection based on expert views in other groups. Following round 2, no new outcome measures will be considered, and the consensus definition (see WP4) will be applied.

The steering committee will be informed of scores and analysis between rounds, with any issues or conflicts being addressed.

### WP4: Consensus Definition and Meeting

#### Consensus Definition

In line with a previously proposed definition by Williamson et al [13], our consensus definition has been discussed and agreed upon by the steering committee and will be applied in round 2 of the Delphi study.

- “Consensus in” (should be included in COS): 70% or more participants scoring the outcome “7 to 9” AND fewer than 15% scoring it “1 to 3”
- “Consensus out” (should not be included in COS): 50% or less, scoring the outcome as 7 to 9
- No consensus (uncertainty about the importance of outcome): Anything else

The consensus criterion needs to be met for each outcome across the 4 stakeholder groups separately for inclusion or exclusion in the final COS; otherwise, the outcome will be classed as “no consensus” and taken forward for discussion.

The results of the Delphi survey will be discussed at a “final consensus meeting” involving invited participants from the 4 stakeholder groups. Outcomes categorized as “consensus in” across the stakeholder groups during the Delphi will be included in the final COS, and those outcomes categorized as “consensus out” will be excluded. Other outcomes that either have no consensus or partial in or out consensus across the stakeholder groups will be discussed within the consensus meeting, including a round of voting on all debated outcomes. If a final COS cannot be decided by the end of this consensus meeting, further consensus meetings may need to be planned to reach the final consensus.

### Ethics Approval

Ethical approval has been granted from the HRA Wales REC 7 committee for phase 1 and phase 2 of the COS study (19/WA/0314). Written consent is required for all face-to-face interviews or focus groups. Verbal recorded consent from health professionals being interviewed over the telephone is acceptable. Participants in the Delphi survey must register online, and implied consent would be assumed if this process is completed.

### Safety Considerations

Due to the study type and voluntary nature of the survey, with the ability to withdraw participation at any time, there are no perceived risks to participants.

### Follow-up

There is no follow-up as part of this study.

### Data Management and Statistical Analysis

Descriptive statistics will display score distribution within stakeholder groups between Delphi rounds. In addition, an analysis of attrition bias will be undertaken.

### Quality Assurance

All data will be stored securely, and all individuals accessing any of the data will comply with the requirements of the Data Protection Act 2018 and adhere to the General Data Protection Regulation (GDPR) and its statements regarding the collection, storage, processing, and disclosure of any personal information.

### Expected Outcomes of the Study

This research will help standardize outcome definition, collection, measurement, analysis, and interpretation in subsequent studies for cranioplasty. In addition, the output will provide the platform for future patient-reported core outcome measures for use in cranioplasty clinical trials.

### Project Management

There is an international steering committee overseeing the study and a working group meeting regularly and reporting to the steering committee.

## Results

This study commenced in December 2019. Phase 1 has been completed, and phase 2 started in Spring 2022, with completion due at the end of 2022/beginning of 2023. The study length will be 3-4 years in total. In phase 2, each round of the Delphi will be 3-4 weeks in length, with a 3-week analysis period in-between rounds. The consensus meetings will then be held before a final consensus meeting with the key stakeholder groups to finalize the COS.

## Discussion

This COS aims to standardize cranioplasty-specific outcomes that would then be recommended for future use across research and clinical practice. Categorizing outcomes into domains under one of the four core areas of the OMERACT 2.0 filter framework [12] will hopefully ensure a broad and patient-focused range of relevant outcomes. Cranioplasty needs to be viewed in context with the underlying brain injury. Therefore, the outcomes for this COS will be specific to the impact cranioplasty has on patients rather than an extension of outcomes of the underlying brain injury. There is overlap, but cranioplasty-specific outcomes will hopefully improve the understanding of the impact of cranioplasty on the trajectory of recovery for patients following brain injury.

We know of no published COS for cranioplasty following decompressive craniectomy for TBI or stroke. The COS is being developed with key stakeholder groups using a robust, standardized, and transparent methodology. This iterative process focuses on ensuring a clear, nonbias pathway for developing the cranioplasty COS, all detailed in this protocol. The continuing involvement of key stakeholder groups provides the relevance of this research to all groups involved and, hopefully, makes it accepted as helpful research in the future.

## Abbreviations

COMET: Core Outcome Measures in Effectiveness Trials
COS: core outcome set
OMERACT: Outcome Measures in Rheumatology
TBI: traumatic brain injury

## Data Availability

Data sharing does not apply to this article as no data sets were generated or analyzed as this is a protocol.
